# Personalized, Music-Based Rhythmic Auditory Stimulation for Exercise Promotion of Older Adults

**DOI:** 10.1093/geroni/igaf122.4385

**Published:** 2025-12-31

**Authors:** Kyoung Shin Park, Bryan Montero Herrera, Brittany Armstrong, Jiyeong Hong, Jeongwoon Kim, Jasmin Hutchinson, David Williams, Jennifer Etnier

**Affiliations:** Emory University School of Medicine, Atlanta, Georgia, United States; University of North Carolina at Greensboro, Greensboro, North Carolina, United States; University of North Carolina at Greensboro, Greensboro, North Carolina, United States; University of North Carolina at Greensboro, Greensboro, North Carolina, United States; Emory University School of Medicine, Atlanta, Georgia, United States; Springfield College, Springfield, Massachusetts, United States; Brown University School of Public Health, Providence, Rhode Island, United States; University of North Carolina at Greensboro, Greensboro, North Carolina, United States

## Abstract

Older adults’ low rate of moderate-to-vigorous physical activity (MVPA) poses a public health crisis. This clinical trial (NCT06496425, NCT06364189) aimed to test the effects of personalized, music-based rhythmic auditory stimulation (RAS) on MVPA over 6 months in older adults. Community-dwelling, low-active older adults (N = 59, Age=70.4±4.4 years, 81% female) were randomized into exercise only (EX) or exercise with RAS (MEX) groups. Both groups received an identical exercise program with aerobic and strength training over 6 months with bimonthly decreases in supervised group sessions from 3 to 1, then 0 days/week. The MEX group was trained to exercise in sync with the tempo of RAS. Weekly MVPA volume was assessed using a waist-worn triaxial accelerometer for 7 days at baseline and on a monthly basis. The data were fitted to a nonlinear mixed-effects model while controlling for covariates (age, sex, race, body mass index). Results indicate that MVPA followed a nonlinear trajectory over time with a significant quadratic effect (p<.001), indicating MVPA increased in the initial months with supervision and decreased during the unsupervised months in both groups. The MEX group had higher MVPA in month 1 (p=.004) and month 2 (p<.017) before exhibiting a steeper decline in the following months (B=–0.127, p=.028) with a distinct cubic pattern of change (B = 0.15, p=.031) compared to the EX group. Random effects indicated substantial individual variability in both baseline MVPA and time slopes. These findings suggest that RAS can successfully increase MVPA through supervised training, but independent adherence to MVPA may require additional strategies.

